# Evaluation of Probiotic and Antidiabetic Attributes of *Lactobacillus* Strains Isolated From Fermented Beetroot

**DOI:** 10.3389/fmicb.2022.911243

**Published:** 2022-06-14

**Authors:** V. B. Chandana Kumari, Sujay S. Huligere, Ramith Ramu, Shrisha Naik Bajpe, M. Y. Sreenivasa, Ekaterina Silina, Victor Stupin, Raghu Ram Achar

**Affiliations:** ^1^Department of Biotechnology and Bioinformatics, School of Life Sciences, JSS Academy of Higher Education and Research, Mysore, India; ^2^Department of Biotechnology, Sri Dharmasthala Manjunatheshwara College (Autonomous), Ujire, India; ^3^Department of Studies in Microbiology, University of Mysore, Mysore, India; ^4^Department of Human Pathology, I.M. Sechenov First Moscow State Medical University (Sechenov University), Moscow, Russia; ^5^Institute of Biodesign and Modeling of Complex Systems, I.M. Sechenov First Moscow State Medical University (Sechenov University), Moscow, Russia; ^6^Department of Hospital Surgery 1, N.I. Pirogov Russian National Research Medical University (RNRMU), Moscow, Russia; ^7^Division of Biochemistry, School of Life Sciences, JSS Academy of Higher Education and Research, Mysore, India

**Keywords:** α-glucosidase, α-amylase, fermented beetroot, lactic acid bacteria, probiotics

## Abstract

Fermented foods are sources of functionally salient microbes. These microbes when ingested can regulate biomolecule metabolism which has a plethora of health benefits. Lactic acid bacteria species (LABs) isolated from fermented beetroot were biochemically characterized and validated using 16s rRNA sequence. Also, an *in vitro* assay was conducted to confirm the probiotic activity of the isolates. The cell-free supernatant (CS), cell-free extract (CE), and intact cell (IC) were evaluated for α-glucosidase and α-amylase inhibition. The six isolates RAMULAB01–06 were categorized to be *Lactobacillus* spp. by observing phenotypic and biochemical characters. Molecular validation using 16S rDNA sequencing, followed by homology search in NCBI database, suggested that the isolates are >95% similar to *L. paracasei* and *L. casei*. Also, isolates exhibited probiotic potential with a high survival rate (>96%) in the gastrointestinal condition, and adherence capability (>53%), colonization (>86%), antibacterial, and antibiotic activity. The safety assessments expressed that the isolates are safe. The α-glucosidase and α-amylase inhibition by CS, CE, and IC ranged from 3.97 ± 1.42% to 53.91 ± 3.11% and 5.1 ± 0.08% to 57.15 ± 0.56%, respectively. Hence, these species have exceptional antidiabetic potential which could be explicated to its use as a functional food and health-related food products.

## Introduction

Diabetes mellitus (DM) is an impaired metabolic syndrome of carbohydrates and lipids resulting in hyperglycemia. In DM, impaired insulin secretion by pancreatic beta cells and its deficiency renders body to assimilate circulating glucose and eventually leading to insulin resistance (Cerf, 2013). α-glucosidase and α-amylase are the enzymes found on the cells that line the intestine. To control diabetes, the inhibition of α-glucosidase and α-amylase delays the absorption of glucose, leading to a slow surge in postprandial blood glucose (Bashary et al., 2019; Hedrington and Davis, 2019).

The role of gut microbiota in the onset and progression of diabetes has been studied extensively; hence, gut health monitoring could be used as a complementary approach for diabetes risk assessment (Qin et al., 2012). An increasing amount of research suggests that consuming *Lactobacillus* spp. or its fermentation products may help people with diabetes (Li et al., 2014; Dang et al., 2018). Antioxidant and α-glucosidase inhibitory activity are reliable approaches for screening probiotics from traditional fermented vegetable products (Chen et al., 2014). The α-glucosidase enzyme activity lowering ability exerted by *Lactobacillus* spp. *in vitro* is by delaying the carbohydrate metabolism (ingestion and absorption), thus causing hypoglycemia, improving glucose equilibrium, pancreatic function, and insulin resistance, and alleviating oxidative damage (Li et al., 2016). The gut colonization of *Lactobacillus* spp. is necessary to manage diabetes. In this regard, LABs need to possess an innate ability of acid bile stability, digestive enzyme resistance, augmentation of food compound solubility, restoration of mucosal integrity, and fabrication of vitamins and enzymes to be effective antidiabetic agents (Fijan, 2014; Terpou et al., 2019). In addition, LABs should possess antibiotic sensitivity, the ability to populate the human gastrointestinal tract (10^8^-10^9^ CFU/mL), adhere to intestinal epithelial cells, and possess antimicrobial activity (de Melo Pereira et al., 2018).

Fermented foods are extensively consumed all around the world. Fermentation is a traditionally majorly used process that is applied for the preservation of foods and for the improvement of their organoleptic properties. It is a spontaneous process generally conducted in a conducive environment, allowing native lactic acid bacteria (LAB) to grow optimally in the fermenting foods. Customarily, from ages fermented vegetables are processed and included in daily life (Lorn et al., 2021; Szutowska and Gwiazdowska, 2021). Consumption of vegetables which are high in antioxidant components and other nutritional components provides a variety of health benefits to humans. According to recent scientific studies, supplementing meals with fermented foods can help to reduce the health risks connected with DM (Srihari et al., 2013; Zulkawi et al., 2018). The LAB isolated from fermented food sources is known for its gut microbiome modulations, decreasing low-grade inflammation, reducing glucose in the bloodstream, and demonstrating antidiabetic effects (Gulnaz et al., 2021; Youn et al., 2021). Beetroot (*Beta vulgaris* L. sub sp. *vulgaris*), known as red beet, is a palatable taproot. Consumption of beetroot enriches cardiovascular health, increases plasma nitrite concentration, reduces blood pressure, enhances exercise performance, and possesses antioxidant and anticancerous properties (Klewicka et al., 2009; Wylie et al., 2013).

Consumption of beetroot juice improves stimulus onset and a signaled response in type-2 diabetes (Gilchrist et al., 2014; Jones et al., 2019). Lacto-fermented beetroot juice improves metabolic activity, diminishes food intolerance, induces the production of betanidin and isobetanidin with antioxidant potential, and increases shelf life of the food source (Czyzowska et al., 2006; Klewicka et al., 2009). Water-soluble betalains (pigment) have been proven to be antioxidant, anti-inflammatory, hepatoprotective, and anticancerous agents (Georgiev et al., 2010). In addition, it is also used as food colorant owing to the presence of intense pigments. Fresh and dried beetroot samples enriched with *Lactobacillus plantarum* Bl3 help in increasing betalains and polyphenols and expressed antioxidant activity (Barbu et al., 2020).

Around the world, there are several fermented food products that offer a plethora of health benefits mostly meat- or milk-based products. Fermented vegetables have health benefits since they are high in carbohydrates, vitamins, and minerals, as well as lack dairy allergies. As a result, expanding the range of commercially available products with probiotic potential is important. The identification and characterization of strains obtained from various fermented vegetable food products may aid in our understanding of their probiotic characteristics and their capabilities. Using the probiotic potential, LAB isolated from fermented vegetables with antidiabetic potential not only improves the gut health but also decreases hyperglycemia. With this background, the objectives of the present study are to identify LABs from fermented beetroot with probiotic potential capable of inhibiting α-glucosidase and α-amylase.

## Materials and Methods

### Materials

The chemicals required for this study were obtained from HiMedia Laboratories Pvt. Ltd., Mumbai, India. The pathogens (*Escherichia coli* MTCC 4430*, Bacillus subtilis* MTCC 10403*, Micrococcus luteus* MTCC 1809*, Pseudomonas aeruginosa* MTCC 424, and *Salmonella enterica typhimurium* MTCC 98) were obtained from Microbial Type Culture Collection and Gene Bank (MTCC), Chandigarh, India.

#### Preparation of Sample

Fresh beetroot (1 kg) obtained from the local market, Mysore, was washed with warm water (32.5 °C). The outer layer was peeled, rinsed again in tap water, and cut into 1/2 to 3/4 inch wedges. Beetroot (250 g) and salt (25 g) were mixed and kept to ferment in a glass jar (four batches) for 4–6 days. LAB strains were isolated using pooled samples from these batches.

#### Isolation

The fermented beetroot samples were taken on the second, fourth, and sixth days for the isolation of LAB strains using the modified method outlined by Ozgun and Vural (2011). The stock cultures were stored (15% glycerol, −20°C) for further study.

### Biochemical Assays of LAB

The preliminary assessment of LAB identification was undertaken as mentioned in Bergey's manual of determinative bacteriology criteria. After incubation at different temperatures (4, 10, 37, 45, and 50°C), there was a visible growth in the MRS broth. The isolated bacteria were assessed for salt tolerance in MRS broth containing 2, 3, 7, and 10% NaCl at 37°C for 48 h. Further, the carbohydrate fermentation using 10 saccharides and the pH tolerance using different pHs, such as 2, 4, 6, and 7.4 of the isolated LAB in MRS broth, were evaluated (Cowan, 1948).

### Cell Preparation

Isolates grown in MRS broth (24 h) were centrifuged at 10,000 rpm (10 min, 4°C). The pellets were collected after washing with phosphate-buffered saline (PBS) pH 7.4. Further, the cells were adjusted to 1 × 10^8^ CFU/mL and incubated for 18–24 h. The assays were performed using the bacteria incubated for 0 and 24 h on MRS agar after serial dilution.

### Probiotic Properties

#### Acid Bile Salt Tolerance

The LAB isolates were evaluated for acid and oxgall salt tolerance as described previously with slight adaptations (Somashekaraiah et al., 2019). In brief, 0.3 and 1% of oxgall salt were prepared in MRS broth and adjusted to pH 2. About 100 μl of LAB isolates was inoculated and incubated (37°C). Enumeration of the samples was performed at 0, 2, and 4 h after inoculation. The cell suspension from each culture attained in the assays was counted on by plating onto MRS agar (24 h, 37°C). The following formula was used to calculate the survival rate (%):


Survival rate(%) ={Biomass at time (t)/Biomass at initial time(0)}×100

#### Determination of Cell Surface Hydrophobicity

The cell surface hydrophobicity provides information on the interaction between xylene (polar solvent) and the bacteria. It was carried out in accordance with the procedure outlined (Pieniz et al., 2015), and the results were calculated using the below equation:


$$\begin{array}{l} \text{Hydrophobicity}\left(\text{H}\%\right)=\left[\left(\text{Ao}-\text{A}\right)/\text{Ao}\right]\;\times 100 \end{array}$$

where H% = cell surface hydrophobicity (%),

Ao = initial absorbance at 600 nm, and

A = final absorbance of the aqueous phase.

#### Phenol Tolerance Assay

MRS broth containing 0.4% phenol was used to evaluate the viability and survival rate of the LAB isolates (10^8^ CFU/mL) as per the procedure defined previously (Jena et al., 2013). In brief, the 0- and 24-h incubated bacterial samples were checked for cell viability and survival rate by serial dilution and were calculated by the colony count method.

#### Autoaggregation and Coaggregation Assay

The method autoaggregation was performed as described by Li et al. (2020) with slight modification. The 18-h culture was collected by centrifugation at 10,000 rpm (3 min), washed, and re-suspended in PBS to obtain 10^8^ CFU /mL cells. This cell suspension was incubated (37°C), and the upper layer was evaluated at 0, 2, 4, 6, 10, and 24 h using a spectrophotometer at an absorbance of 600 nm. The rate of autoaggregation was calculated as follows:


$$\begin{array}{l} \text{Autoaggregation}\%=\left[\left(\text{Ao}-{\text{A}}_{\text{t}}\right)/\text{Ao}\right]\times 100 \end{array}$$

where A_0_ = absorbance at time 0= 0 h and A_t_ = absorbance at time t = 2, 4, 6, 10, or 24 h.

For coaggregation, the sample was prepared similar to that of autoaggregation assay. LAB isolates and the five pathogenic strains (*Escherichia coli* MTCC 4430, *Bacillus subtilis* MTCC 10403*, Micrococcus luteus* MTCC 1809*, Pseudomonas aeruginosa* MTCC 424, and *Salmonella enterica typhimurium* MTCC 98) each 1 ml were mixed and incubated for 2 h (37 °C). After the incubation, the sample's absorbance was measured at 600 nm and percentage of coaggregation was computed as below:


$$\begin{array}{l} \left[\left({\text{A}}_{\text{LAB}}+{\text{A}}_{\text{Path}}\right)-{\text{A}}_{\text{mix}}\right]/\left({\text{A}}_{\text{LAB}}+{\text{A}}_{\text{Path}}\right)\times 100, \end{array}$$

where A_LAB_ + A_Path_ signifies the LAB and pathogen combination absorbance at time 0 h, and A_mix_ signifies the absorbance at time 2 h.

#### Simulated Gastric Juice Tolerance Assay

To simulate gastric and intestinal juice, pepsin (3 g/L) and trypsin (1 g/L) were dissolved in PBS at pH 3 and pH 8, respectively. The simulated juice was sterilized by passing through a 0.22-μm filter membrane. The cultured cells (10^8^ CFU/mL) were suspended into simulated gastric juice and incubated at 37°C for 0, 1, and 3 h in 5% CO_2_ incubator. The cells were then added to simulated intestinal juice (9 mL) and incubated at 37°C for 1, 3, 5, and 8 h after 3 h in 1 mL of simulated gastric juice. The selected strain gastrointestinal tolerance was assessed using viable colony counts (Mantzourani et al., 2019). After serial dilution and incubation at 37°C for 24 h, a viable count of the LAB isolates was measured by spread plate technique on MRS agar. The following equation was used to compute the survival rate:


$$\begin{array}{l} \text{Survival rate}\%=\left(\text{log cfu }{\text{N}}_{1}\right)/\left(\text{log cfu }{\text{N}}_{0}\right)\times \;100 \end{array}$$

where N_1_ = the total viable count of LAB strains after treatment and N_0_ = the total viable count of LAB strains before treatment (Bao et al., 2010).

#### Antibiotic Sensitivity

As per the Clinical and Laboratory Standards Institute (2018), the LAB isolates were evaluated for antibiotic susceptibility using disk diffusion method. The LAB cultures (10^8^ CFU/mL) were spread plated on MRS agar and incubated. The plates were then inoculated with antibiotic disks and incubated at 37°C for 24 h. The antibiotic susceptibility of the isolates was evaluated using chloramphenicol (30 mcg/disk), gentamicin (10 mcg/disk), clindamycin (2 mcg/disk), ampicillin (10 mcg/disk), kanamycin (30 mcg/disk), tetracycline (30 mcg/disk), vancomycin (30 mcg/disk), erythromycin (15 mcg/disk), streptomycin (100 mcg/disk), rifampicin (5 mcg/disk), methicillin (5 mcg/disk), azithromycin (15 mcg/disk), and cefixime (5 mcg/disk). The antibiotic zone scale was used to determine the diameter of the zone of inhibition (CLSI scale), using which the results were interpreted as susceptible, moderately susceptible, or resistant (Auta and Hassan, 2016; Chang, 2018).

#### Antibacterial Activity

The agar well-diffusion method was used to test the antibacterial activity of LAB isolates against 10 pathogenic bacteria viz., *Escherichia coli* (MTCC 443)*, Bacillus subtilis* (MTCC 10403)*, Micrococcus luteus* (MTCC 1809)*, Pseudomonas aeruginosa* (MTCC 424), *Salmonella enterica typhimurium* (MTCC 98), *Bacillus cereus* (MTCC 1272)*, Staphylococcus aureus* (MTCC 1144)*, Klebsiella pneumonia* (MTCC 10309)*, Pseudomonas fluorescens* (MTCC 667), *and Klebsiella aerogenes* (MTCC 2822) as per the method described by Yadav et al. (2016).

### Molecular Characterization of LAB

Molecular identification of the isolated LABs was performed using the universal primers 27F-5'AGAGTTTGATCCTGGCTCAG3' and 1492R-5'GGTTACCT TGTTACGACTT3' to amplify 16s rRNA sequence, following the procedure described previously (Boubezari et al., 2018). The isolates on the basis of their probiotic potential were subjected to DNA isolation and amplification. The amplified PCR products were sequenced, and the homology search was conducted by BLAST (basic local alignment search tool). The sequences were submitted to the GenBank sequence database, and accession numbers were obtained (Boubezari et al., 2018; Dhameliya et al., 2020).

#### Sequencing Homology Search and Phylogenetic Analysis

Using MEGA X (Version 10.2.4, CA, USA), the sequenced 16s rRNA region of six LAB isolates from the current study was used to create a phylogenetic tree. The maximum likelihood phylogenetic tree with 1,000 bootstrap consensus was conducted. The Tamura–Nei model was the best fit model (Tamura and Nei, 1993). The Neighbor-Join and BioNJ algorithms were applied to a matrix of pairwise distances to automatically generate the initial tree(s) for the heuristic search (Kumar et al., 2018).

### *In vitro* Adhesion to Chicken Crop Epithelial Cells

Chicken crop epithelial cells were collected according to the methodology described by Somashekaraiah et al. (2019) to test the LAB isolate's capacity to connect to epithelial cells under *in vitro* conditions. The 100 μl of chicken crop epithelial cells (1 ×10^6^ cells/mL) and 10 μL of LAB isolates (10^8^ CFU/mL) were well-mixed and incubated (30 min, 37°C). Then, the non-adherent bacteria were eliminated by centrifuging at 3,000 rpm (3 min). Furthermore, the pellet was washed twice and re-suspended in sterile PBS (100 μL) and subjected to crystal violet staining later for the microscopic observation.

### *In vitro* Adhesion to Buccal Epithelial Cells

The methodology described in previous study was used to test the LAB isolate capacity to connect buccal epithelial cells under *in vitro* conditions (Somashekaraiah et al., 2019). In brief, buccal epithelial cells were used to study LAB adherence to their surfaces. Cells were collected from a healthy volunteer. For the reduction in normal microbiota, the mouth of the volunteer was washed twice with sterile saline solution, and then, the buccal cells were collected. The obtained epithelial cells were washed twice with saline solution. Then, the cells were subjected to centrifugation at 5,000 rpm for 2 min. The supernatant was decanted, and the pellets were washed thrice in sterile PBS before being suspended in saline. About 400 μL of diluted buccal epithelial cells (3 × 10^6^ cells) was mixed with 100 μL LAB isolates (10^8^ CFU/mL) and was incubated for half an hour. The cells were stained with crystal violet and tested for LAB adherence to the buccal epithelial cells.

### HT 29 Cell Culture and Growth Conditions

Adhesion capacity of the six isolates to human colon cancer cell lines (HT-29) was evaluated according to previously mentioned methodology (Fonseca et al., 2020). HT29 cell lines (passage #123–130) were acquired from the National Center for Cell Science (NCCS), Pune, India. HT29 was grown in DMEM (25 mM) without sodium pyruvate, but with GlutaMAX (Gibco, UK) at 37°C in an incubator with 5% CO_2_ and 95% air. A total of 10 % (v/v) FBS (Gibco, UK), and 100 μg/mL penicillin and streptomycin were added to the media (Verhoeckx et al., 2015). About 1 × 10^5^ cells/mL of HT-29 cells was sub-cultured in six-well-culture plate and cultivated at 37°C in a humidified CO_2_ atmosphere until they reached 80 percent confluence in cell medium. The culture medium was changed on alternate days.

Isolates grown in MRS broth for 16 h at 37°C were used for the adhesion assay. At a concentration of 10^8^ CFU/mL, cells were re-suspended in DMEM media and washed twice with PBS. About 1 mL of bacterial suspension was added to each well and incubated for 30 and 60 min at 37°C in a 5% CO_2_ environment. Then, 1 mL of 0.1% Triton-X solution (in PBS) was used to lyse the cells and remove non-adherent bacterial cells and rinsed thrice with 1000 ul of PBS. After 10 min at 37°C, the solution containing liberated bacterium cells was serially diluted and plated on MRS agar. The plates were incubated for 24 h at 37°C. The percentage ratio between the initial number of bacteria seeded and the counts (CFU/mL) after the washing steps was used to measure adhesion ability. The experiments were carried out in pairs and three times each (Fonseca et al., 2020).

### Hemolytic Activity

The hemolytic activity of the isolates was evaluated using the procedure given in the previous study (Yasmin et al., 2020). The streak plate isolates (on blood agar medium with 5% (w/v) sheep blood) were taken. The lysis of red blood cells in the media around the colonies was observed for hemolytic activity. No zone around the colonies was considered as safe classified as γ-hemolysis, the green zones around colonies as α-hemolysis, and a clear zone as β-hemolysis.

### DNase Activity

To test for DNase enzyme production, the LAB isolates were streaked onto a deoxyribonuclease (DNase) agar medium. The plates were tested for the presence of DNase activity zone after 48 h of incubation at 37°C. A prominent reddish zone around the colonies indicated positive DNase activity (Shuhadha et al., 2017).

### Antioxidant Assay

The antioxidant assay of the isolates was measured using ABTS and DPPH radical-scavenging activities described by Xing et al. (2006) and Yang et al. (2020), respectively.

### Preparation of Intact Cells and Intracellular Cell-Free Extracts

The selected LAB isolates were incubated for 18 h at 37°C and extracted by centrifugation at 2,000 rpm for 15 min. The cell-free supernatant (CS) was filtered through a 0.22-μm filter, whereas the intact cells (IC) in the pellet were washed thrice and suspended in PBS (pH 7.4) and adjusted to 1 × 10^8^ CFU/mL. For the preparation of cell-free extract (CE), the extract was sonicated to break the cell wall of 1 × 10^8^ CFU/mL cells for 15 min at 3 s pulse with 1-min interval on the ice bath. Further, centrifugation was performed at 8,000 rpm for 20 min to collect the supernatant. The latter was sterilized through a 0.22-μm filter to remove bacterial cells (Chen et al., 2014).

### Inhibitory Assay for the Carbohydrate Hydrolyzing Enzymes (α-Glucosidase and α-Amylase)

The α-glucosidase inhibition activity was performed as described (Kim et al., 2011). The test samples (CS, CE, and IC) were mixed with 50 mM of potassium phosphate buffer (pH 6.8, 700 μ L) and incubated for 10 min. α-glucosidase (100 μL, 0.25 U/mL) enzyme was added to this mixture and pre-incubated for 15 min at 37°C. Then, 100 μL of 5 mM p-nitrophenol-D-glucopyranoside (pNPG) substrate was added and incubated at 37°C for 30 min before stopping the enzymatic reaction with 1,000 μL of 0.1 M Na_2_CO_3_. As a standard reference, acarbose was used (positive control). The 4-nitrophenol absorption was measured at 405 nm using a microplate reader (Multiskan FC Microplate Photometer, Thermo Fisher Scientific, France), and the inhibition of α-glucosidase activity of LAB strains was calculated as below:


$$\begin{array}{l} \text{Inhibition of}\alpha -\text{glucosidase}\%=\left(1-{\text{A}}_{\text{S}}/{\text{A}}_{\text{C}}\right)\text{x}100 \end{array}$$

where A_S_ = absorbance of the reactants with the sample and A_C_= absorbance of the reactants without the sample.

Subsequently, the α-amylase inhibition assay was carried out as described (Kwon et al., 2006). Porcine pancreatic amylase was used in the inhibition assay. In brief, 500 μl of CS, CE, and IC, and 500 μl of 0.1 M PBS (pH 7.4) containing α-amylase enzyme (0.5 mg/mL) were pre-incubated for 10 min at 25 °C. In addition, each tube received 500 μl of 1% starch solution in 0.1 M PBS (pH 7.4). The reaction solutions were then incubated for 10 min at 25°C before being stopped with 1.0 mL of 3, 5-dinitrosalicylic acid reagent. After 5 min in a boiling water bath, the test tubes were cooled to room temperature, diluted with 10 mL of distilled water, and the absorbance was measured at 540 nm. The percentage of inhibition exerted by the bacterial strain on α-amylase activity was obtained as defined for α-glucosidase.

### Statistical Analysis

All of the tests were carried out in triplicates. The standard deviation is shown in error bars on the graphs. One-way analysis of variance was used to examine the data (ANOVA). Differences were considered substantial at *p* ≤ 0.05.

## Results

### Preliminary Characterization of LAB Strains

According to the phenotypic characterization, a total of 20 strains were isolated from fermented beetroot samples with six isolates identified as *Lactobacillus* spp. All the strains were bacilli, gram-positive, and catalase-negative. The tested isolates confirmed that they could survive at 37°C but not at 4°C and 50°C. The isolates were able to tolerate 2 and 3% salt concentration, whereas the optimum growth was at pH 7.4 and showed only mild growth at the other pH tested. The isolates were hetero-fermentative, producing only acid but no gas from glucose, according to biochemical analysis. Sucrose, maltose, lactose, and galactose were the carbohydrates fermented by the isolates (Table 1).

**Table 1:** Phenotypic features and fermentation ability of LAB strains obtained from fermented beetroot samples.

	**Isolates**					
**Tests**	**RAMULAB01**	**RAMULAB02**	**RAMULAB03**	**RAMULAB04**	**RAMULAB05**	**RAMULAB06**
Gram staining	Positive					
Catalase	Negative					
Morphology	Rod	Rod	Rod	Short rod	Short rod	Rod
**Growth at different temperature**						
4	-	-	-	-	-	-
10	+	+	+	+	-	-
37	+	+	+	+	+	+
45	-	-	-	-	-	-
50	-	-	-	-	-	-
**Growth at different NaCl concentration**						
2%	+	+	+	+	+	+
3%	+	+	+	+	+	+
7%	+	**-**	**-**	**-**	**-**	**-**
10%	**-**	**-**	**-**	**-**	**-**	**-**
**Carbohydrate fermentation**						
Glucose	+	+	+	+	+	+
D-Xylose	-	+	-	-	-	-
L-xylose	-	-	-	-	-	-
Sucrose	+	+	+	+	+	-
Mannitol	-	-	-	-	-	-
Maltose	+	+	+	-	+	+
Lactose	+	+	+	+	+	+
Galactose	+	+	+	+	+	+
Arabinose	-	-	-	-	-	-
Starch	-	-	-	-	-	-
**Growth at different pH**						
2	+	+	+	+	+	+
4	+	+	+	+	+	+
6	+	+	+	+	+	+
7.4	+	+	+	+	+	+
**IMViC test**						
Indole test	-	-	-	-	-	-
Methyl Red test	+	+	+	+	+	+
Voges-Proskauer test	-	-	-	-	-	-
Citrate utilization test	-	-	-	-	-	-
Starch hydrolysis test	-	-	-	-	-	-
Gelatin liquification test	-	-	-	-	-	-

*+ Indicates presence and - indicates absence*.

### Probiotic Properties

#### Acid Bile Salt Tolerance

**Figure 1:**
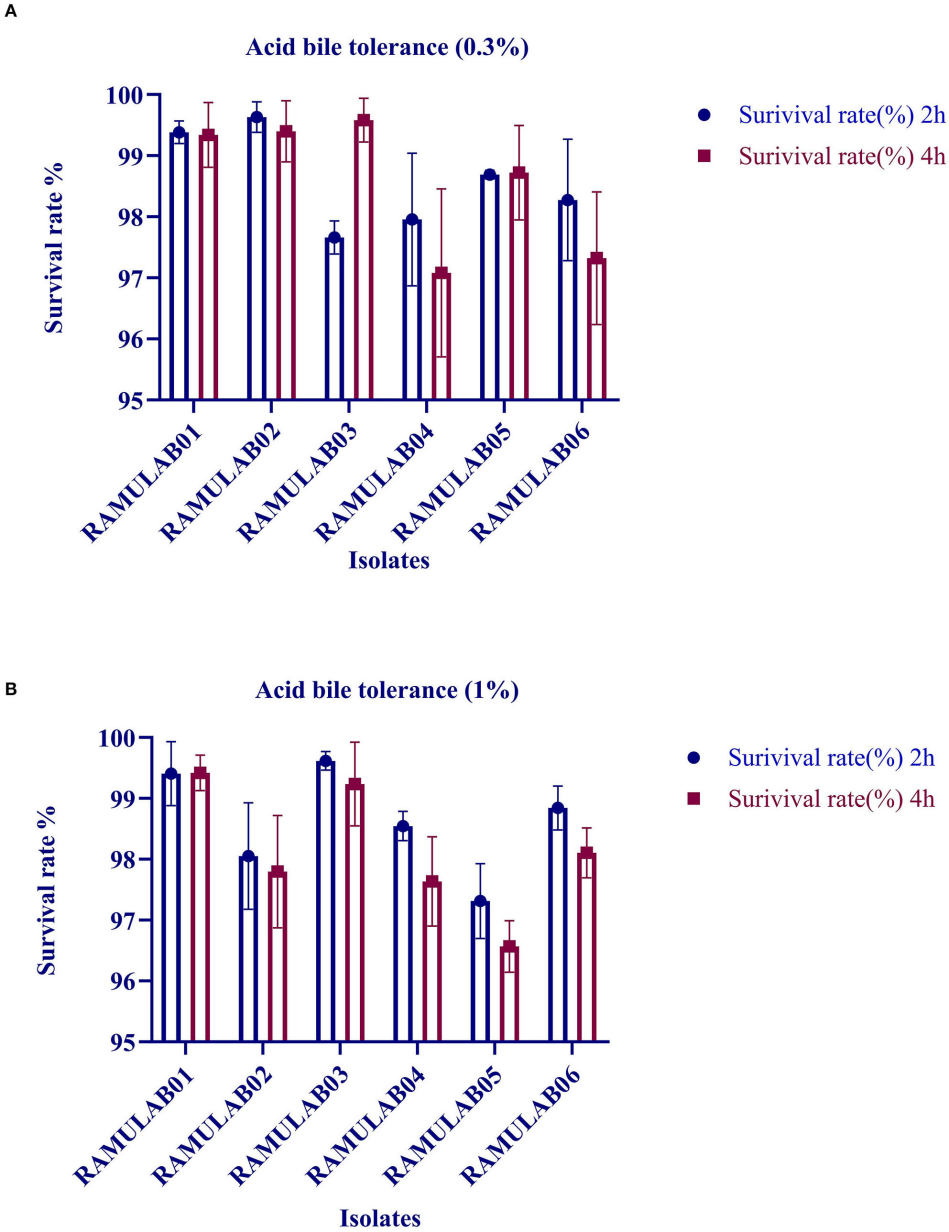
Survival rate of LAB strains obtained from fermented beetroot samples in an acidic pH 2.0 and varied **(A)** 0.3% and **(B)** 1% oxgall concentration at 37°C.

The acid and bile tolerance is conducted to study the survival rate of the isolates at extreme conditions. After 2 and 4 h of incubation at 37°C, the survival rates of all the isolates are presented in Figures 1A,B, respectively. The six isolates RAMULAB01–06 demonstrated a survival rate (%) varying from 97 to 99% at 0.3%, and 96% and 99% at 1% oxgall concentration (*p* ≤ 0.05) under acidic pH conditions.

#### Cell Surface Hydrophobicity

The cell surface hydrophobicity is based on the hydrophobic components of the outer membrane present in the LABs. Hydrophobicity for LAB is to determine the cell attachment to the epithelial cells and colonization of the isolates. The hydrophobicity of the isolates to the xylene (non-polar solvent) ranged from 54.64 to 77.88%. All the isolates had hydrophobicity above 60%. Among all the isolates tested, RAMULAB05 exhibited maximum hydrophobicity of 77.88% and a minimum hydrophobicity was expressed by the RAMULAB06 strain with 54.64% (Table 2).

**Table 2:** Cell surface hydrophobicity of six isolates expressed in percentage.

**Isolates**	**Hydrophobicity (%)**
RAMULAB01	61.90 ± 0.68
RAMULAB02	66.56 ± 0.00
RAMULAB03	68.65 ± 1.09
RAMULAB04	67.61 ± 2.45
RAMULAB05	77.88 ± 4.89
RAMULAB06	54.64 ± 3.60

*The values are expressed as mean ± SD*.

#### Phenol Tolerance Assay

After 24 h of incubation, the viable count of the LAB isolates was determined by inoculating them in an MRS agar plate. The isolates had the ability to tolerate phenol at a concentration of 0.4% as shown (Table 3). There was no apparent change in the rate of growth observed even after 24 h of incubation. The viable count of the isolates varied between 7.46 and 7.87 log CFU/mL with RAMULAB02 showing an optimal tolerance of 7.87 ± 0.51 log CFU/mL (*p* ≤ 0.05) viable counts.

**Table 3:** Survival rate of isolates in gastrointestinal juice and phenol tolerance.

**Survival rate (%)**	**Gastric juice (%)**		**Intestinal juice (%)**				**Phenol tolerance (Log CFU/mL)**	
**Isolates**	**1 h**	**3 h**	**1 h**	**3 h**	**5 h**	**8h**	**0 h**	**24h**
RAMULAB01	99.58 ± 0.21	99.20 ± 0.40	99.87 ± 0.04	99.78 ± 0.08	98.98 ± 0.31	98.32 ± 0.47	7.57 ± 0.00	7.56 ± 0.01
RAMULAB02	99.74 ± 0.14	99.59 ± 0.13	99.92 ± 0.06	99.65 ± 0.33	98.58 ± 1.20	97.63 ± 1.25	7.61 ± 0.01	7.87 ± 0.51
RAMULAB03	99.44 ± 0.14	98.88 ± 0.52	99.71 ± 0.24	99.25 ± 0.42	98.78 ± 0.28	97.83 ± 0.79	7.46 ± 0.06	7.49 ± 0.12
RAMULAB04	99.67 ± 0.14	99.42 ± 0.52	99.92 ± 0.24	99.31 ± 0.42	98.63 ± 0.28	97.64 ± 0.79	7.49 ± 0.00	7.47 ± 0.12
RAMULAB05	99.54 ± 0.18	99.12 ± 0.08	99.90 ± 0.07	99.60 ± 0.23	99.24 ± 0.28	98.85 ± 0.16	7.61 ± 0.01	7.80 ± 0.56
RAMULAB06	99.19 ± 0.20	99.86 ± 0.26	99.58 ± 0.08	99.27 ± 0.22	98.99 ± 0.09	98.99 ± 0.16	7.46 ± 0.06	7.77 ± 0.60

*The values are expressed as mean ± SD and significant (p ≤ 0.05)*.

#### Autoaggregation and Coaggregation Assay

**Figure 2:**
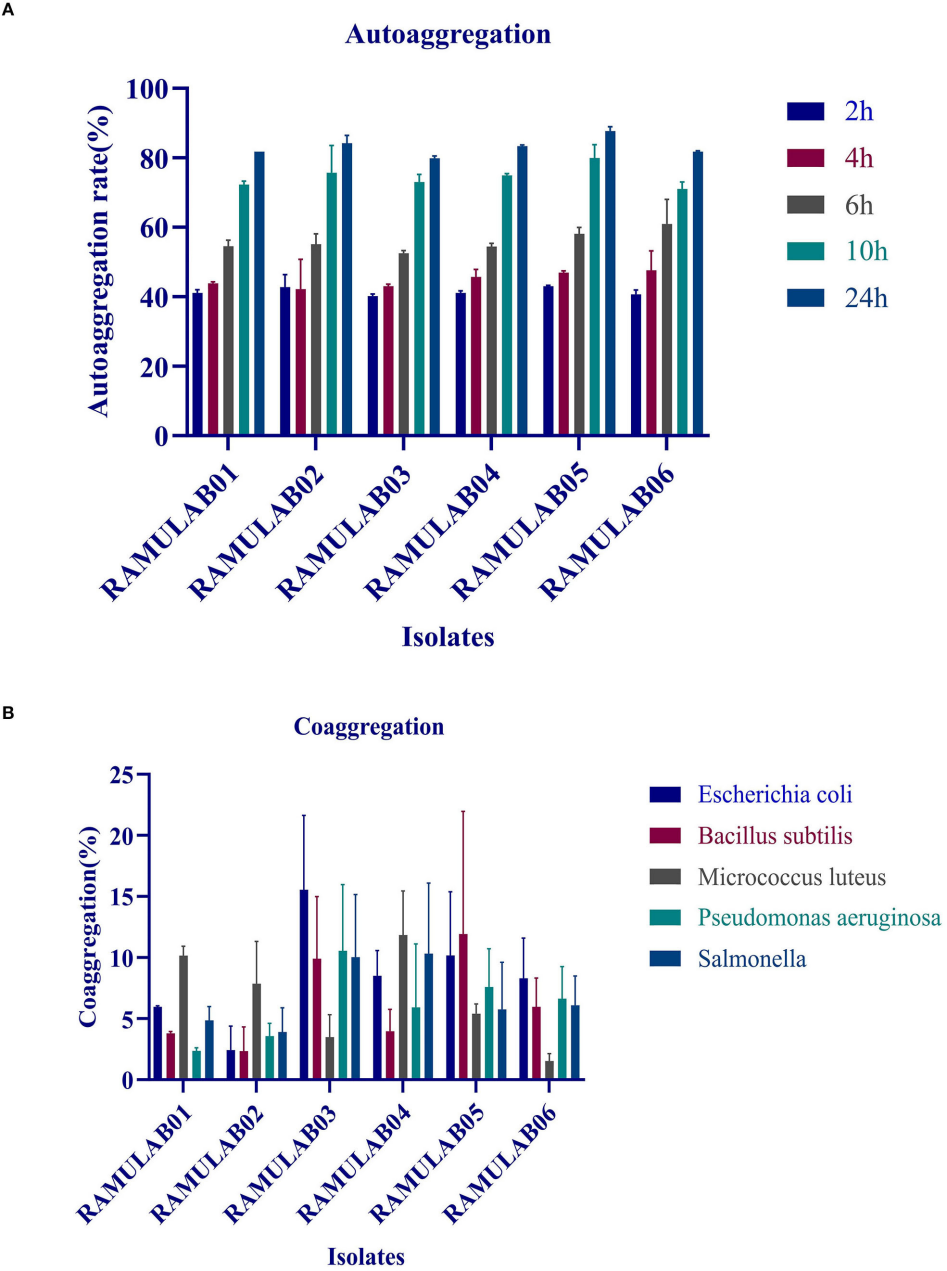
**(A)** Autoaggregation (%) of LAB strains at different time intervals at 37°C; **(B)** coaggregation (%) of LAB strains after incubation of 2 h at 37°C.

The autoaggregations of probiotics are vitally important for the colonization and protection of bacteria. In our study, the autoaggregation of the LAB strains increased as the time progressed. At 24 h, all of the isolates displayed a high autoaggregation activity of more than 79% (Figure 2A), with RAMULAB05 exhibiting the highest percentage of 87.68%.

The coaggregation of the pathogens with the tested isolates was moderate as shown in Figure 2B. Higher coaggregation ability was observed in all the isolates with *M. luteus* except for the isolate RAMULAB03. Among all the isolates, RAMULAB06 had a higher coaggregation with *E. coli, B. subtilis, P. aeruginosa*, and *Salmonella* sp.

#### Simulated Gastrointestinal Juice Tolerance Assay

**Figure 3:**
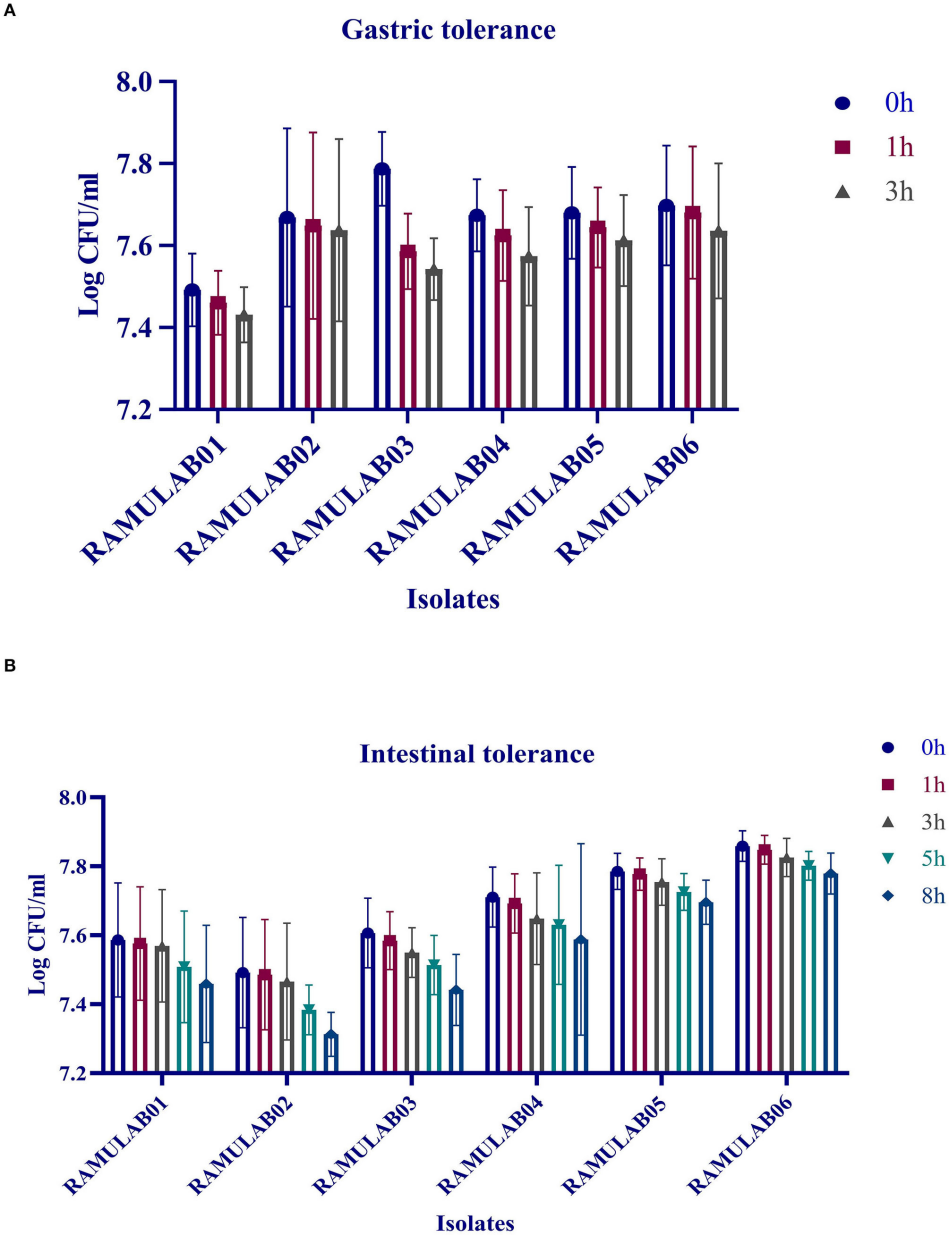
Gastric **(A)** and intestinal juice **(B)** tolerance (log CFU/mL) of LAB strains after incubation for various time intervals at 37°C. The values are expressed as mean ± SD.

The gastrointestinal juice tolerance test of the isolates was performed to evaluate the ability to tolerate the gastrointestinal environment. All the six isolates in the present study showed optimum growth under the digestive environment with a gradual reduction in the growth with prolonged incubation (Figure 3). All the six isolates demonstrated a survival rate of above 97% (Table 3).

#### Antibiotic Sensitivity

The isolates were tested against 13 different antibiotics to determine the profile of antibiotic resistance and compared with that of the reference standard chart to obtain the results because one of the criteria for a microbe to qualify as a probiotic properties includes its sensitivity to antibiotics. The six isolates were sensitive to chloramphenicol, gentamicin, clindamycin, ampicillin, tetracycline, erythromycin, streptomycin, rifampicin, and azithromycin. However, they exerted resistances against kanamycin, vancomycin, methicillin, and cefixime (Table 4 and Supplementary Table 1).

**Table 4:** Antibiotic susceptibility test of the isolates representing resistance and sensitive.

**Isolates**	**C**	**GEN**	**CD**	**AMP**	**K**	**TET**	**VA**	**E**	**STR**	**RIF**	**MET**	**AZM**	**CEF**
RAMULAB01	S	S	S	S	R	S	R	S	S	S	R	S	S
RAMULAB02	S	S	S	S	R	S	R	S	S	S	R	S	S
RAMULAB03	S	S	S	S	R	S	R	S	S	S	R	S	S
RAMULAB04	S	S	S	S	R	S	R	S	S	S	R	S	S
RAMULAB05	S	S	S	S	R	S	R	S	S	S	R	S	S
RAMULAB06	S	S	S	S	R	S	R	S	S	S	R	S	S

*The symbols R and S represent resistance and sensitive, respectively*.

#### Antibacterial Activity

The isolates were tested against diverse foodborne pathogens for antimicrobial activity. It is noteworthy to mention that all of the isolates tested in our study had antibacterial activity against all of the strains. The zone of inhibition ranged from 6 to 20 mm. All of the isolates had a good antibacterial efficacy against the foodborne pathogens *M. luteus* and *P. aeruginosa*. The isolate RAMULAB01 revealed a good antimicrobial activity against all the pathogens except *B. cereus, Salmonella, K. pneumonia, and K. aerogenes*, for which it showed an inhibitory effect of moderate strength. The isolate RAMULAB06 presented minimal activities against all the tested microorganisms expect *S. aureus, P. aeruginosa*, and *M. Luteus* pathogens, for which it showed a mild inhibitory effect (Table 5).

**Table 5:** Antibacterial activity of isolated *Lactobacillus* strains.

**Isolates**	***B. cereus***	***S. aureus***	***Salmonella***	***E. coli***	***P. aeruginosa***	***K. pneumoniae***	***M. luteus***	***B. subtilis***	***P. florescens***	***K. aerogenes***
RAMULAB01	+	++	+	++	++	+	+++	+	++	+
RAMULAB02	+	++	+	+	+++	-	++	++	++	++
RAMULAB03	-	++	+	+	++	++	+++	+	++	+
RAMULAB04	-	++	+	+	++	+	+++	+	++	+
RAMULAB05	+	++	-	++	++	+	+++	++	++	-
RAMULAB06	+	+	+	+	++	-	+++	++	++	++

*Symbols represent zones of inhibition in mm: (-): no inhibition; (+): weak inhibition (<7); (++): good inhibition (9–15); and (+++): strong inhibition (>15)*.

### Molecular Characterization and Phylogenetic Analysis

**Figure 4:**
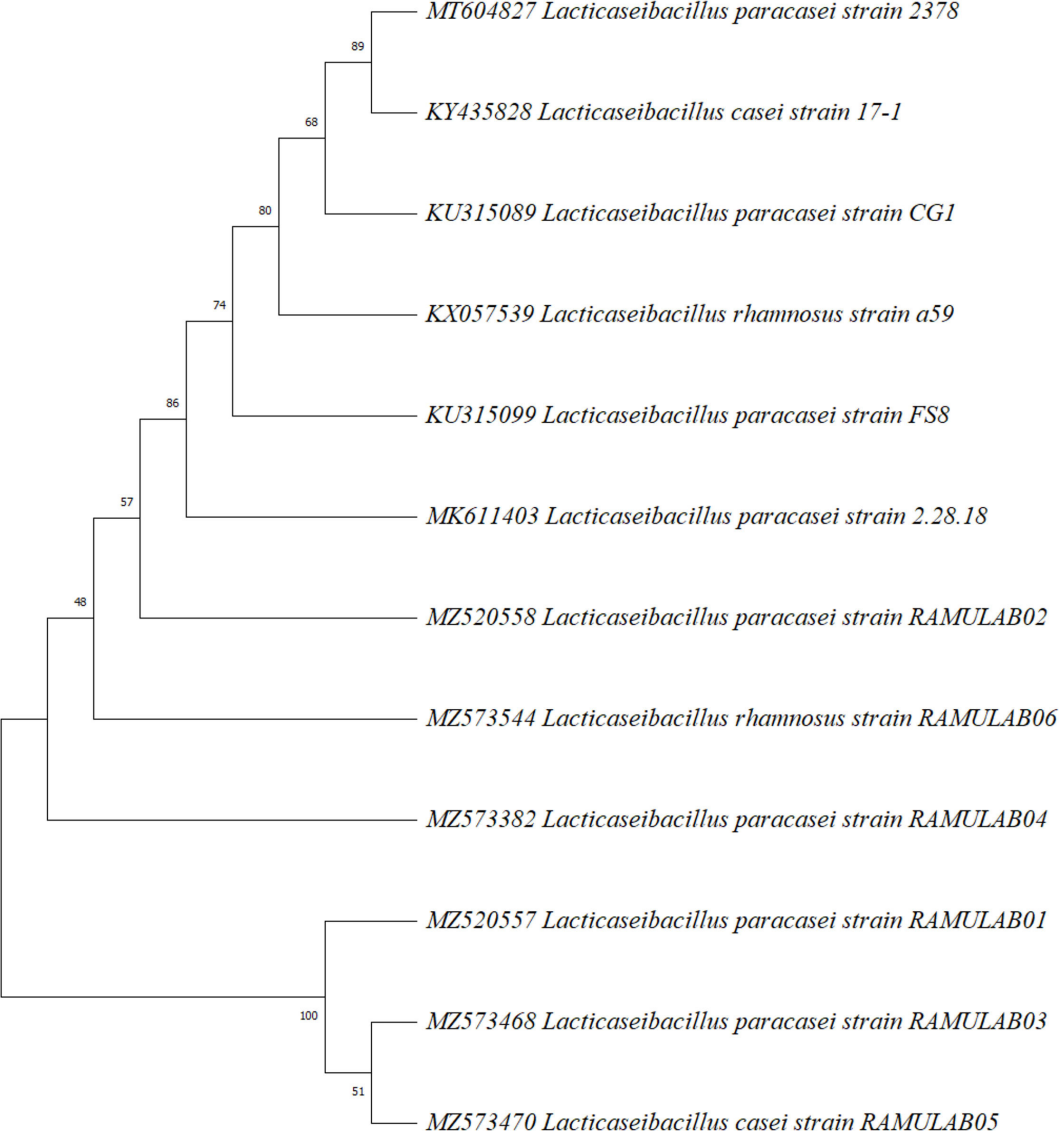
Phylogenetic tree of LAB isolates (RAMULAB01–RAMULAB06) from fermented beetroot samples based on maximum likelihood bootstrap analysis of 16S rDNA.

The biochemically characterized LAB isolates were amplified. The sequence length varied from 730 bp to 1,348 bp. The homology search of the sequences RAMULAB01–RAMULAB04 had >95% similarity to *Lactobacillus paracasei*. The sample RAMULAB05 and RAMULAB06 had a similarity >95% to *Lactobacillus casei* and *Lactobacillus rhamnosus*, respectively, thus validating the isolates sequenced. Of the six isolates, RAMULAB5 was dissimilar to the other isolates as shown in Figure 4.

### Adhesion Ability to Chicken Epithelial Cells, Buccal Epithelial Cells, and HT 29 Cell Lines

**Figure 5:**
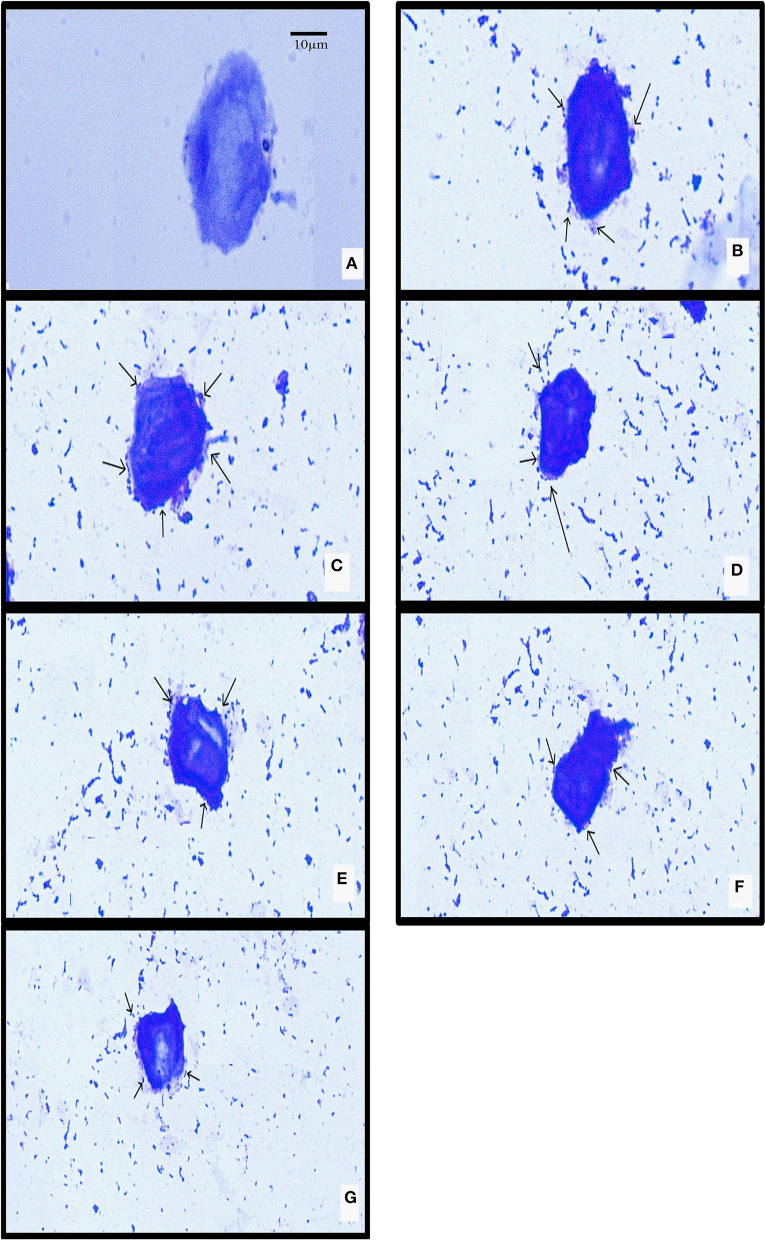
LAB strain adhesion to chicken crop epithelial cells observed under a light microscope. **(A)** Chicken crop epithelial cells (Control). The adhesion to chicken epithelial cells to isolates **(B)** RAMULAB01, **(C)** RAMULAB02, **(D)** RAMULAB03, **(E)** RAMULAB04, **(F)** RAMULAB05, and **(G)** RAMULAB06.

**Figure 6:**
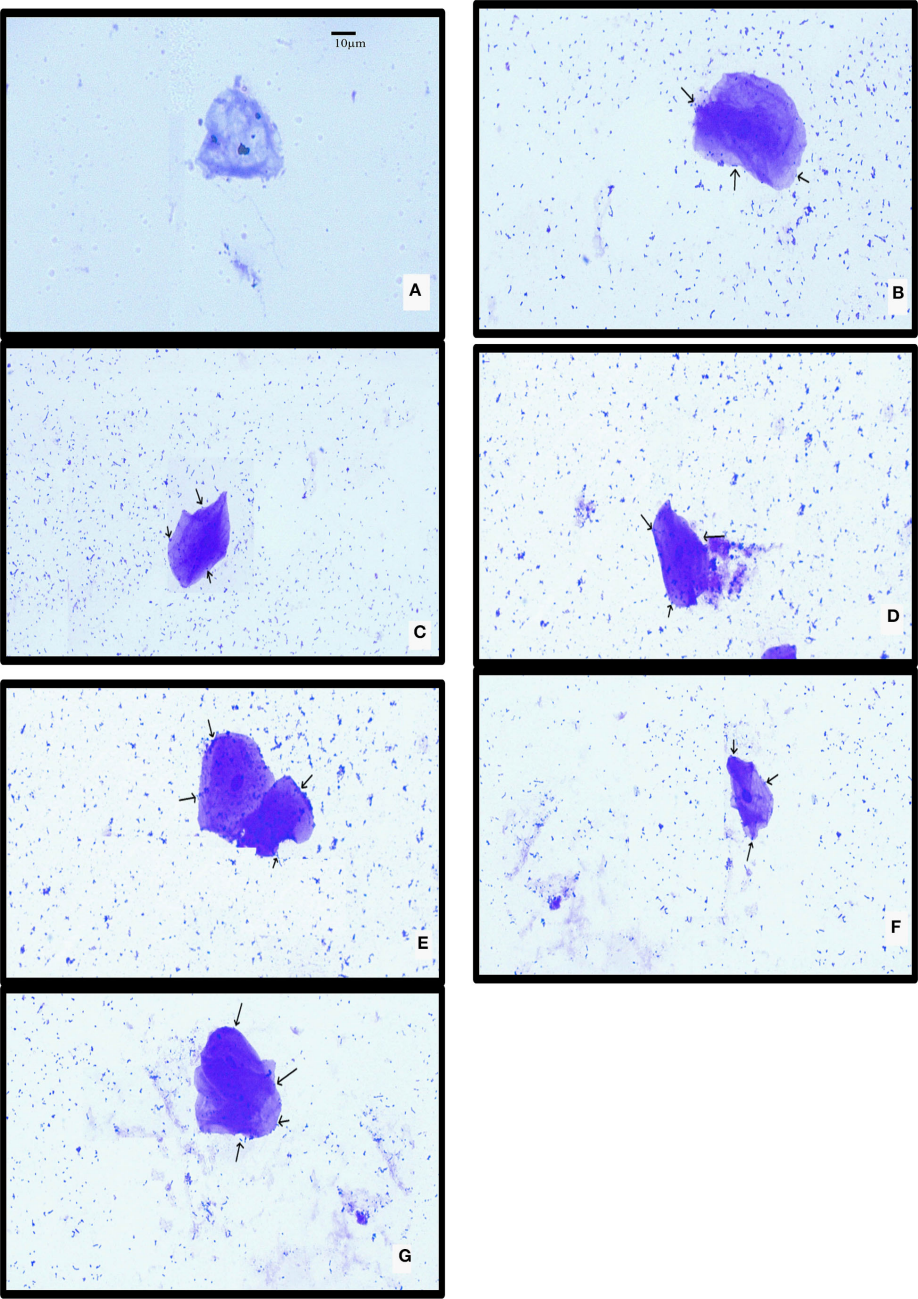
LAB strain adhesion to buccal epithelial cells observed under a light microscope. **(A)** Buccal epithelial cells (Control). **(B)** The adhesion to buccal epithelial cells to isolates **(B)** RAMULAB01, **(C)** RAMULAB02, **(D)** RAMULAB03, **(E)** RAMULAB04, **(F)** RAMULAB05, and **(G)** RAMULAB06.

The isolated chicken epithelial cells were investigated, and the adhesion capacity was determined to be 30–45 bacterial cells per epithelial cell with the least being 14–18 cells (Figure 5). RAMULAB06 had the best adhesion, whereas RAMULAB03 had the least. For buccal epithelial cells (Figure 6), all of the strains had a similar ability as the chicken epithelial cells. The isolate's adherence to the HT-29 cells was expressed above 95% as shown in Table 6.

**Table 6:** Adhesion expressed in percentage of the isolates adhering to HT-29 cells.

**Isolate**	**Adhesion (%)**
RAMULAB01	96.37 ± 1.57
RAMULAB02	96.10 ± 0.13
RAMULAB03	96.68 ± 1.28
RAMULAB04	95.53 ± 1.99
RAMULAB05	95.69 ± 2.83
RAMULAB06	96.53 ± 1.53

*The values are expressed as mean ± SD*.

### Safety Assessments of the Isolates

Safety assessment was performed for the LAB isolates by measuring their hemolytic activity. All the isolates in this study showed no zone of lysis around the colonies, which is classified as γ-hemolysis and considered as safe. Similarly, DNAse activity was also a measure to determine the probiotics safety formulation. In our study, all the isolates demonstrated no zone of inhibition confirming that the isolates are non-pathogenic and regarded safe for development of probiotic formulation.

### Antioxidant Assay

**Figure 7:**
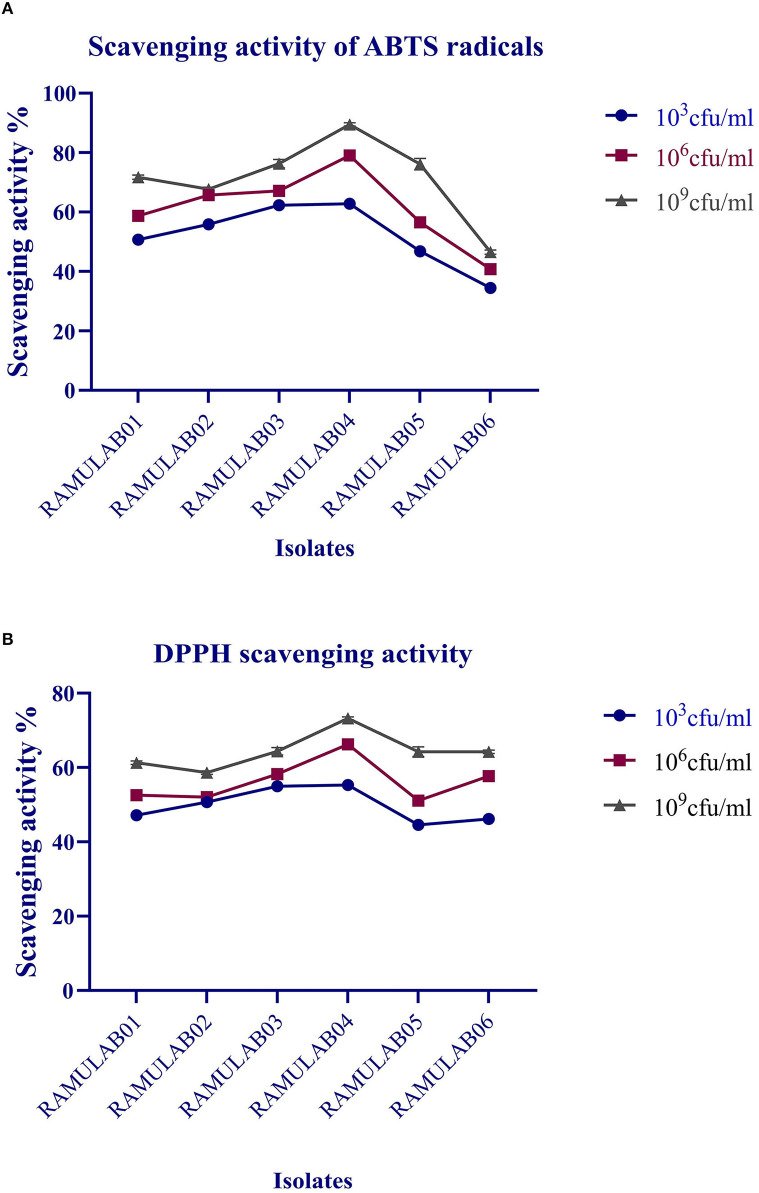
Scavenging activity of ABTS radicals **(A)** and DPPH free radical-scavenging activity **(B)** of the isolates. The values are expressed as mean ± SD.

The radical-scavenging activity for ABTS radicals exhibited by the isolates ranged from 16.53 to 89.49%, with RAMULAB04 showing 89.49%, which was the highest scavenging activity, and RAMULAB06 showing 34.46%, which was the lowest (Figure 7A). The isolates evaluated for DPPH free radical-scavenging activity revealed a higher scavenging activity as observed by the increase in the number of cells expressed as CFU/mL (Figure 7B). At 10^9^ CFU/mL, RAMULAB04 had the highest radical-scavenging activity (73.23%), followed by RAMULAB03, RAMULAB05, RAMULAB01, RAMULAB02, and RAMULAB06.

### Inhibitory Assay for the Carbohydrate Hydrolyzing Enzymes

**Figure 8:**
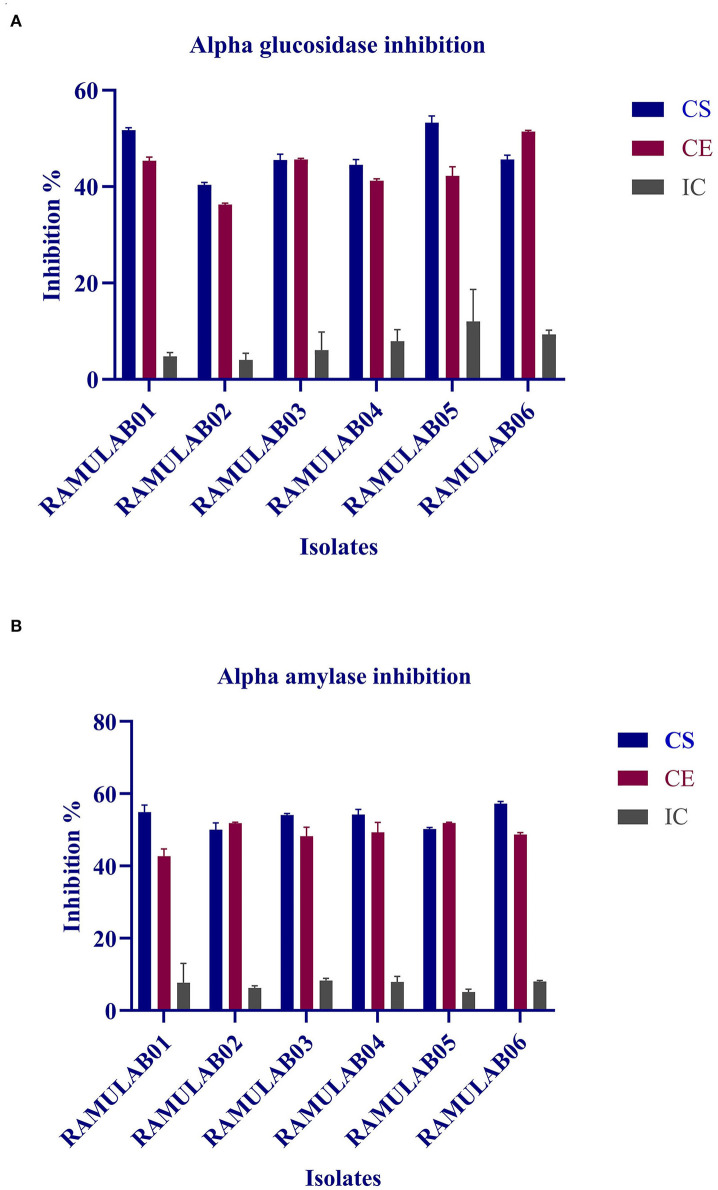
α-glucosidase **(A)** and α-amylase **(B)** inhibitory activity of the various extract (CS, CE, and IC) isolates. The values are expressed as mean ± SD.

Both α-glucosidase and α-amylase, the key enzymes of carbohydrate metabolism, were efficiently inhibited by CE and CS from all the isolates. The inhibitory potential of CS, CE, and IC on α-glucosidase ranged from 4.0 to 53.3% and α-amylase from 6.2 to 57.2% (Table 7). Overall, among the various extracts (CE, CS, and IC) tested, IC showed the least inhibition compared with that of the supernatant and pellets (Figures 8A,B).

**Table 7:** α-glucosidase and α-amylase inhibitory activity of the isolates.

**Isolates**	α**-Glucosidase inhibition (%)**			α**-Amylase inhibition (%)**		
	**CS**	**CE**	**IC**	**CS**	**CE**	**IC**
RAMULAB01	51.70 ± 0.54	45.45 ± 0.7	04.78 ± 0.81	54.91 ± 1.87	42.74 ± 1.97	07.67 ± 5.26
RAMULAB02	40.45 ± 0.53	36.32 ± 0.35	03.98 ± 1.42	50.03 ± 1.89	51.84 ± 0.30	06.21 ± 0.63
RAMULAB03	45.51 ± 1.21	45.60 ± 0.35	06.13 ± 3.67	54.08 ± 0.36	48.23 ± 2.45	08.25 ± 0.60
RAMULAB04	44.45 ± 1.08	41.20 ± 0.39	07.85 ± 2.37	54.21 ± 1.38	49.26 ± 2.74	07.91 ± 1.50
RAMULAB05	53.31 ± 1.39	42.18 ± 1.89	11.95 ± 6.70	50.22 ± 0.42	51.93 ± 0.24	05.09 ± 0.79
RAMULAB06	45.6 ± 0.92	51.37 ± 0.34	09.34 ± 0.92	57.15 ± 0.56	48.68 ± 0.47	08.03 ± 0.31

*The values are expressed as mean ± SD and significant (p ≤ 0.05)*.

## Discussion

The fermented food products are gaining popularity in recent times owing to its remarkable medicinal potential. Fermented foods play an important role in the diet, and various health benefits have been associated with the fermentation process over time. *Lactobacillus* spp. have been exploited with an extensive antiquity in food fermentation that have created a new field and design of a functional foods (Lorn et al., 2021). In this study, the viability of the LAB isolates was evaluated at different temperatures, salt tolerance, and acid bile tolerance. Compared to previous studies, the viability rate at various temperatures, salt, and bile tolerance was higher than 80% (Yadav et al., 2016). It is widely known that the digestion of consumed food takes up to 3 h in the abdominal environment (pH 2) and 3–8 h in the intestine (pH 8) that constantly subjects them to extreme conditions. In order for a drug to survive these conditions, they should ensure tolerance to the bile and gastrointestinal juices that contain several enzymes that otherwise degrade the molecules rendering them inactive (Begley et al., 2005; Gu et al., 2008).

In this study, the six isolates showed remarkable survival rates at low pH. Also, in the presence of oxgall, it was found that the survival rate was above 96% for 0.3% and 1% oxgall concentration despite a low pH even after incubation for 4 h. In addition, >98% survival rate was observed for all six isolates tested for bile tolerance, which was higher than that reported in the previous studies by Zielińska et al. (2014) for *L. brevis* O22 (survival rate% 97.8; pH 2.5) and gastrointestinal tolerance reported by Romero-Luna et al. (2020) *L. paracasei* CT12 (survival rate at 30 min in gastric was 97.19 ± 3.9%, and intestinal environment was 42.12 ± 9.6). A similar finding on pickled vegetable strains exhibited a high probability of survival during bile salt stress, according to a recent study. Nonetheless, our findings are consistent with the previous findings that indicated the number of cells in all of the strains studied reduces after 24 and 48 h of incubation. The gastrointestinal microorganisms are capable of producing phenol and the other toxic metabolites liberated by certain digestive processes. Therefore, any microorganism that can survive these conditions can qualify to possess probiotic potential (Padmavathi et al., 2018; Yasmin et al., 2020). Thus, isolates that are tolerant to phenol prove that they can survive gastrointestinal environment. The prevention of pathogen colonization is a major criterion for any microorganism to be a potential probiotic. In this study, the hydrophobicity, autoaggregation, and coaggregation were evaluated to understand this aspect of the LAB isolates. While autoaggregation is the process of binding of the colonies of the same group of microorganisms, hydrophobicity is a parameter that helps them bind to the intestinal layer and coaggregation is the adhesion between different strains at the intercellular level. This study demonstrated that the autoaggregation was over 80% along with varying levels of coaggregation. This phenomenon helps in the maintenance of a healthy ecosystem in the intestine (Nikolic et al., 2010; Pieniz et al., 2015; Li et al., 2020). *Lactobacillus* spp. that has the greatest ability to attach is thought to have the best immune modulation. As a result, determining the isolate's adhesion to intestinal epithelium cells aids in determining its ability to colonize the intestine, this in turn avoids pathogen adhesion. Recent investigations have described the capacity of competitive adherence of the *Lactobacillus* spp. to prevent inflammatory consequences while creating a higher state of change in the defense system of the host intestinal epithelial cells (Cammarota et al., 2009; Dhanani and Bagchi, 2013). In this study, the antibiotic susceptibility is assessed that showed that the isolates were resistant against kanamycin, vancomycin, methicillin, and cefixime. These LAB isolates can be recommended for its beneficial effects in improving the intestinal health, especially when LABs are simultaneously administered together with antibiotics, which can prevent infections caused by other pathogens. In this study, all the isolates showed a good antimicrobial activity against *M. luteus* and *Pseudomonas aeruginosa*, which are opportunistic foodborne pathogens. In a similar finding, Jiang et al. (2018) reported that the bacteriocin derived from *L. plantarum* ZJ316 had promising antimicrobial mechanism against *M. luteus* by exhibiting membrane permeabilization activity. *L. rhamnosus*, on the contrary, has shown to disrupt cellular membrane integrity and cause ATP efflux, resulting in pore formation and inhibiting *M. luteus* growth (Li et al., 2005). As per the previous study, *P. aeruginosa* strain inhibiting capability of LAB was proven as the cause for biofilm production of the LAB isolates against the pathogen (Shokri et al., 2017). The DNase test was also performed to check for the pathogenicity of bacteria that produce DNase enzyme resulting in DNA hydrolysis. As a result, the lack of DNase in the isolates tested was confirmed, indicating their potential safety of its use in fermentation. Similarly, a hemolytic experiment revealed no hemolysis, indicating that the isolated LAB strains are safe (Yadav et al., 2016).

The ability of intact cells to scavenge free radicals is linked to the cell surface components of the bacteria. The isolates in this investigation had a higher scavenging activity in agreement with several other previous studies. Excessive generation of free radicals has been associated with diabetes pathogenesis and development (Jaganjac et al., 2013). A vast range of oxygen-centered free radicals and reactive oxygen species are produced by the human body and food systems (Parvez et al., 2006; Wang et al., 2017). The most dangerous reactive oxygen species are hydroxyl and related radicals, which cause oxidative damage to biomolecules. DPPH and ABTS take electrons or hydrogen atoms from antioxidants and transform them into compounds that are irreversibly stable. Previous studies demonstrated that the intact cells from a few LAB isolates, such as *P. pentosaceus* R1 and *L. brevis* R4, displayed a substantially higher ABTS radical-scavenging activity than cell-free extract and supernatant and are in agreement with our findings. NADH, NADPH, antioxidant enzymes, Mn^2+^, bioactive chemicals, and exopolysaccharides are all antioxidant substances found in LAB strains (Jiang et al., 2018).

This study also evaluated the inhibitory potential of the probiotic isolates against the enzymes of carbohydrate metabolism namely, α-glucosidase and α-amylase. Kwun et al. (2020) reported that the MBEL1397 isolated from kimchi demonstrated 3.91% α-glucosidase inhibitory activity, corresponding to approximately 2.3 times higher than that of acarbose. In a similar study performed by Son et al. (2017), *L*. *brevis* KU15006 isolate exhibited 24.11% α-glucosidase inhibitory activity for CS and 10.563% for CE, which fared better than even the commercially available LAB. *L. reuteri*-KX881777, *L. plantarum*-KX881772, and *L. plantarum*-KX881779 isolates reported by Ayyash et al. (2018) revealed that their water-soluble extracts (WSEs) had α-amylase inhibition capability >34% in the exopolysaccharide crude and pure extract from *Lactobacillus* H31 isolated from pickled cabbage, as reported by Huang et al. (2021). Their study showed that pancreatic α-amylase was inhibited by ~89.1 ± 2.59% and 69.2 ± 8.95%, respectively, for the two extracts. From this study, it was elucidated that CS, CE, and IC of the isolates demonstrated inhibition against both the carbohydrates hydrolyzing enzyme (α-glucosidase and α-amylase). The intestinal α-glucosidase and α-amylase inhibition help in the management of postprandial hyperglycemia and control glucose circulating in the blood. In this regard, diabetic complications can be delayed by the inhibitory activity of α-glucosidase and α-amylase (Ramu et al., 2014; Ademiluyi et al., 2015).

## Conclusion

This study constitutes the first-ever research on the fermented beetroot showing its competence for harboring the potential probiotic LAB isolates with antidiabetic properties. The results from this study demonstrated that LABs isolated were safe with properties, such as tolerance to bile salt and acid, gastrointestinal environment, along with remarkable autoaggregation and coaggregation capabilities, hydrophobicity abilities, antibiotic, and antimicrobial activities that are essential criteria for them to qualify as a probiotics. CS and CE of the six isolates had a remarkably high inhibitory activity on α-glucosidase and α-amylase in comparison with that of the IC. Thus, the probiotic microorganism from the fermented beetroot can be suggested as a feasible source for antidiabetic components. The research findings from this study could further support the development and utilization of beetroot as a fermented functional food for their notable probiotic potential.

## Data Availability Statement

The original contributions presented in the study are included in the article/Supplementary Material, further inquiries can be directed to the corresponding author.

## Author Contributions

RR designed the research work. VK and SH performed the research activities. VK, ES, VS, RRA, and SN analyzed the data and validated. RR, VK, and SN wrote the manuscript. RR, VK, SN, and MS edited the manuscript submitted. All authors have given their approval for publication.

## Conflict of Interest

The authors declare that the research was conducted in the absence of any commercial or financial relationships that could be construed as a potential conflict of interest.

## Publisher's Note

All claims expressed in this article are solely those of the authors and do not necessarily represent those of their affiliated organizations, or those of the publisher, the editors and the reviewers. Any product that may be evaluated in this article, or claim that may be made by its manufacturer, is not guaranteed or endorsed by the publisher.
