# Supplementary material for: Evaluation of Probiotic and Antidiabetic Attributes of Lactobacillus Strains Isolated From Fermented Beetroot
Source: Front Microbiol. 2022 Jun 14;13:911243. doi: 10.3389/fmicb.2022.911243 (PMC9237538; doi:10.3389/fmicb.2022.911243)
Supplement: Supplementary file 1 [file Table_1.DOCX]

**Supplimentary Table 1.** Breakpoints of sensitivity/resistance (S/R) in the mm inhibitory zone of respective antibiotics based on CLSI, 2018.

| **Sl.No.** | **Antibiotic** | **the inhibitory zone(S/R mm)** |
| --- | --- | --- |
| 1 | Chloramphenicol (C) | (≥18/≤12) |
| 2 | Gentamicin (GEN) | (≥15/≤12) |
| 3 | Clindamycin (CD) | (≥19/≤14) |
| 4 | Ampicillin (AMP) | (≥17/≤14) |
| 5 | Kanamycin (K) | (≥18/≤12) |
| 6 | Tetracycline (TET) | (≥19/≤14) |
| 7 | Vancomycin (V) | (≥17/≤14) |
| 8 | Erythromycin (E) | (≥23/≤13) |
| 9 | Streptomycin (STR) | (≥15/≤12) |
| 10 | Rifampicin (RIF) | (≥20/≤16) |
| 11 | Methicillin (MET) | (≥22/≤4) |
| 12 | Azithromycin (AZM) | (≥13/≤12) |
| 13 | Cefixime (CEF) | (≥21/≤2) |
